# Comparison of three-dimensional reconstruction and CT-guided Hook-wire segmental resection for pulmonary nodules: a propensity score matching study

**DOI:** 10.1186/s12957-023-03035-4

**Published:** 2023-05-27

**Authors:** Ziqiang Hong, Yingjie Lu, Yannan Sheng, Baiqiang Cui, Xiangdou Bai, Tao Cheng, Xusheng Wu, Dacheng Jin, Yunjiu Gou

**Affiliations:** 1 The First Clinical Medical College, Gansu University of Chinese Medicine, Lanzhou, China; 2 Department of Thoracic Surgery, Gansu Provincial Hospital, Lanzhou, China

**Keywords:** Pulmonary nodules, Three-dimensional reconstruction, Hook wire, Thoracoscopic surgery, Propensity score matching

## Abstract

**Objective:**

To analyze and compare the clinical application value of three-dimensional reconstruction and computed tomography (CT)-guided Hook-wire localization for row lung segment resection of pulmonary nodules.

**Methods:**

Retrospective analysis of the clinical data of 204 patients suffering from pulmonary nodules admitted to the Department of Thoracic Surgery of Gansu Provincial People’s Hospital from June 2016 to December 2022. According to the preoperative positioning method, the group was divided into a 3D reconstruction group (98 cases) and a Hook-wire group (106 cases), respectively. The two groups of patients were propensity score matching (PSM) to compare their perioperative outcomes.

**Results:**

All patients in both groups underwent successful surgeries without perioperative deaths. After PSM, 79 patients were successfully matched in each group. Two cases of pneumothorax, three cases of hemothorax, and four cases of decoupling occurred in the Hook-wire group; no complications of pneumothorax, hemothorax, and decoupling occurred in the 3D reconstruction group. Compared to the Hook-wire group, the 3D reconstruction group has shorter operative time (*P* = 0.001), less intraoperative bleeding (*P* < 0.001), less total postoperative chest drainage (*P* = 0.003), shorter postoperative tube placement time (*P* = 0.001), shorter postoperative hospital stay (*P* = 0.026), and postoperative complications (*P* = 0.035). There was no statistically significant difference between the two groups in terms of pathological type, TNM staging, and number of lymph node dissection.

**Conclusion:**

Three-dimensional reconstruction and localization of pulmonary nodules enables safe and effective individualized thoracoscopic anatomical lung segment resection with a low complication rate, which has good clinical application value.

## Introduction

Pulmonary nodules are focal, round-like, densely increased lung shadows characterized by solid or subsolid imaging and a diameter of ≤ 30 mm [1]. The benign and malignant pulmonary nodules should be differentiated according to the imaging characteristics, and the suspected malignant pulmonary nodules should be removed as soon as possible. With the development of minimally invasive technology and the improvements in surgical instruments, thoracoscopic lung surgery developed rapidly. Compared with traditional thoracotomy, thoracoscopic surgery causes less trauma and results in faster recovery times for patients, making it widely used in clinical practice [2]. However, due to the limitation of small incision in thoracoscopic surgery, the surgeon’s finger cannot fully reach deeper pulmonary nodules within the chest cavity, so the localization of pulmonary nodules is the key to pulmonary nodule surgery. For accurate and rapid localization of pulmonary nodules, CT-guided Hook-wire pulmonary nodule localization method often used clinically, but it is an invasive operation that can lead to complications such as pneumothorax, hemothorax, and decortication [3]. Therefore, the development of safer, more accurate, rapid, and noninvasive methods for localizing pulmonary nodules is an urgent clinical challenge. In this study, we compared the clinical results of CT-guided Hook-wire and 3D reconstructed lung nodule localization methods for thoracoscopic lung segmental resection to investigate the safety and application value of 3D reconstructed lung nodule localization method.

## Material and methods

### Clinical information

The clinical data of 204 patients with pulmonary nodules admitted to the Department of Thoracic Surgery of Gansu Provincial People’s Hospital from June 2016 to December 2022 were included. The inclusion criteria were as follows: (I) chest CT showing pulmonary nodules < 2 cm in diameter and > 50% glassy composition, (II) no distant metastasis of tumor was found on any preoperative examinations, (III) single nodule or multiple nodules with a single operation to treat only the major lesion, and (IV) patients who did not receive neoadjuvant radiotherapy before surgery with complete clinical case information. Exclusion criteria were as follows: (I) poor cardiopulmonary function that could not tolerate surgery and (II) the surgical methods involving lobectomy or wedge resection. All patients included in this study were informed of the advantages and disadvantages of both localization methods, and the lung nodule localization method was selected based on a combination of factors including the location of the patient’s lung nodule, the intention of the patient, and his family members.

This study has been reviewed by the Ethics Committee of Gansu Provincial People’s Hospital, approval number 2023–018. All patients signed the informed consent form for surgery before surgery.

### Pulmonary nodule localization methods

#### Three-dimensional reconstruction of lung nodule localization method

**Fig. 1:**
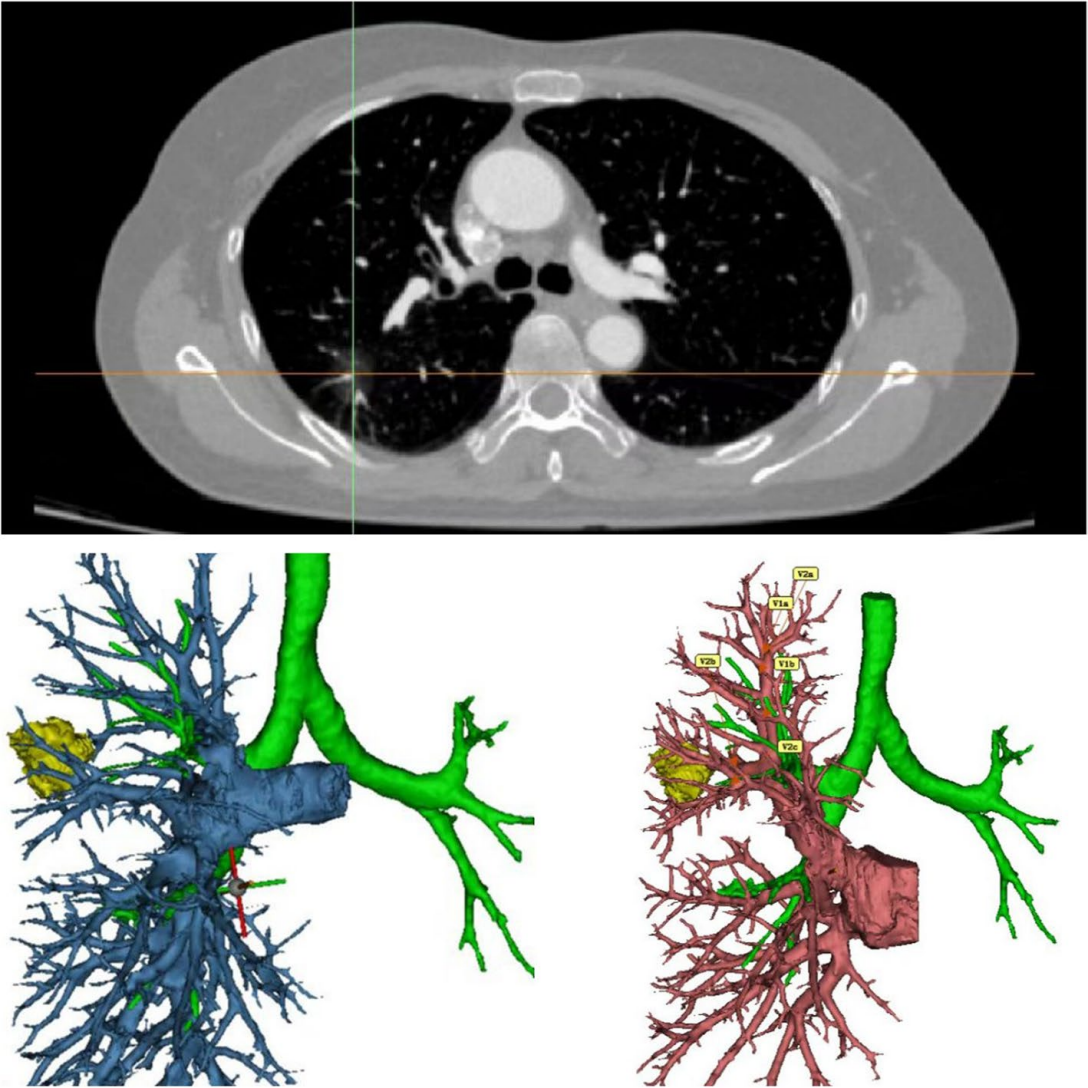
Schematic diagram of 3D reconstruction of lung nodule localization

All patients underwent preoperative enhanced CT (with a scanning layer thickness of 1.0 mm) examination of the chest, and the 3D reconstruction software Mimics Medical 21.0 was applied to store the reconstruction data and construct 3D reconstruction images. Preoperatively, a thoracic surgeon and two radiologists analyzed and created 3D reconstructed image (e.g., Fig. 1) to clarify the affected lung segment and its anatomical relationship with surrounding tissues. We will reconstruct the bronchi, pulmonary arterioles, and nodules in 3D before surgery and marked them with different colors. A sphere was created around the nodule, with a margin of 2 cm from its surface or no less than the diameter of the nodule. The cutting plane and resection area were manually designed according to the lesion location, ensuring that the shortest distance from the lung parenchyma resection margin to the tumor margin was ≥ 2 cm or equal to the maximum tumor diameter. The operator simulated the surgery and developed the best plan based on the 3D reconstructed images.

#### CT-guided Hook-wire lung nodule localization method

**Fig. 2:**
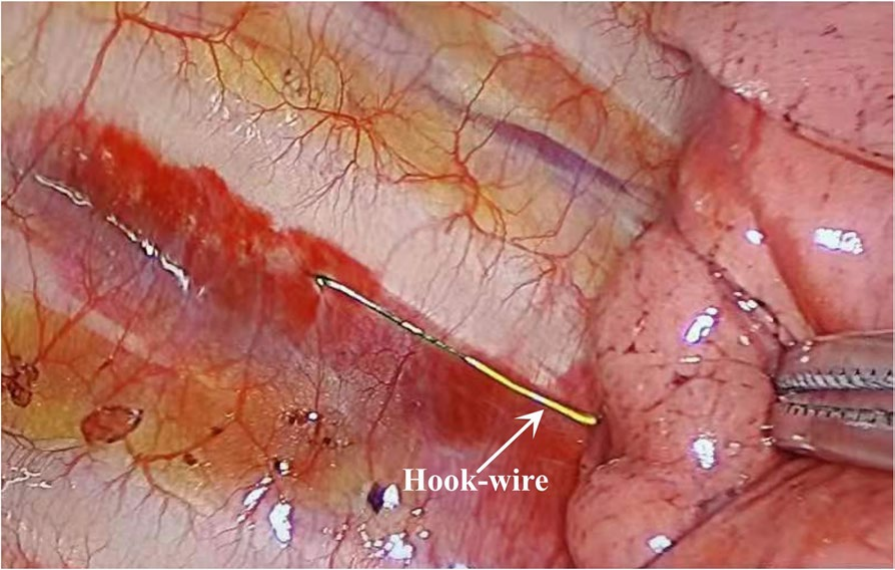
Hook-wire localization of pulmonary nodule

The puncture was scheduled to be performed within 2 h prior to the procedure, and a chest CT scan was first conducted to determine the entry point, depth of entry, and angle of entry. The Hook wire was then punctured next to the pulmonary nodule, the CT scan of the chest is repeated to confirm the puncture position, and the barb is released. After localization, the chest CT was carefully examined to determine the location of the pulmonary nodule intraoperatively based on the position between the nodule and the Hook wire (Fig. 2).

#### Surgical method

General anesthesia was administered along with double-lumen tracheal intubation, healthy side lung ventilation, and a healthy side folding position. In a small incision in the anterior axillary line between the 5th or 6th ribs, about 3 cm long, incision protection sleeve and lumpectomy instruments were placed. In the 3D reconstruction group, the computerized 3D reconstruction image was placed next to the thoracoscopic screen in the operating room. An off-stage assistant rotated, displayed, or hid the 3D reconstruction image according to the operator’s requirements, so that the operator can refer to it in real time for surgery. In the Hook-wire group, the location of the pulmonary nodule was determined intraoperatively based on the position of the locating hook. The bronchus was taken as the core, and the artery was treated first while minimizing damage to intersegmental veins. The bronchus, pulmonary artery, and vein were dissected free from the focal lung segment to a sufficient distance. After clamping the bronchus of the focal lung segment with a cutting occluder, pure oxygen was used to inflate the lung. Confirmation of the correct identification of the bronchus in the target segment was conducted before cutting it off. The lung segment where the lesion is located was inflated and then ventilated with one lung, with atrophy used to form a boundary between the inflated lung tissue and the surrounding area. The lesion was excised using a linear cutter closure and sent for frozen pathology. Lymph node sampling was performed at stations N1 and N2 for adenocarcinoma in situ or microinvasive adenocarcinoma, while systemic lymph node dissection was carried out for other types of cancer. If positive lymph node cryopathology was present, conversion to lobectomy with systemic lymph node dissection was conducted.

### Statistical analysis

A 1:1 PSM analysis was performed using SPSS 26.0 software with the caliper value setting to be 0.02, and the matching factors included sex, age, body mass index (BMI), smoking history, nodule size, and nodule depth. Continuous variables were expressed as mean ± standard deviation ($\stackrel{\mathrm{-}}{\text{x}}$ ± s), and *t*-test for two independent samples was used for comparison between groups, while categorical variables were expressed as frequencies and percentages (%), and chi-square test or Fisher test was used for comparison between groups. Two-sided *P*-value less than 0.05 was considered to be statistically significant difference.

## Results

### PSM results

After PSM on the 98 patients in the reconstruction group and the 106 patients in the Hook-wire group, 79 patients in each group were finally successfully matched. Before matching, the baseline characteristics of the two groups were unbalanced, with statistically significant differences in age (*P* = 0.026), and after matching, the differences in the six confounding factors were not statistically significant between the two groups (all *P* > 0.05), and the baseline characteristics of the patients were balanced, as shown in Table 1. The specific distribution of the types of lung segments to be resected is listed in Table 2.

**Table 1:** Comparison of baseline information before and after propensity score matching between the two groups (cases/$\stackrel{\mathrm{-}}{\text{x}}$ ± *s*)

Characteristics	Before PSM			After PSM		
	**3D reconstruction group (*n* = 98)**	**Hook-wire group (*n* = 106)**	***P***	**3D reconstruction group (*n* = 79)**	**Hook-wire group (*n* = 79)**	***P***
Sex			0.709			0.425
Male	58 (59.2)	60 (56.6)		45 (57.0)	40 (50.6)	
Female	40 (40.8)	46 (43.4)		34 (43.0)	39 (49.4)	
Age (years)	56.91 ± 5.68	58.65 ± 5.49	0.027	56.67 ± 5.83	57.82 ± 4.98	0.184
BMI (kg/m^2^)	23.54 ± 2.36	23.27 ± 2.51	0.435	23.73 ± 2.27	23.24 ± 2.61	0.207
Smoking history			0.678			0.484
Yes	27 (27.6)	32 (30.2)		21 (26.6)	25 (31.6)	
No	71 (72.4)	74 (69.8)		58 (73.4)	54 (68.4)	
Nodule size (mm)	10.08 ± 3.10	9.89 ± 2.00	0.591	10.29 ± 3.30	9.96 ± 1.98	0.449
Nodular depth (mm)	17.15 ± 3.80	16.38 ± 3.57		17.44 ± 3.65	16.63 ± 3.34	0.147

Abbreviations: *BMI* body mass index, *LUL* left upper lobe, *LLL* left lower lobe, *RUL* right upper lobe, *RML* right middle lobe, *RLL* right lower lobe, *Nodular depth* depth from the visceral pleural surface

**Table 2:** Lung subsegmental resection sites

Type of planned segmentectomy	3D reconstruction group (*n* = 79)	Hook-wire group (*n* = 79)
Right lung		
S1	6 (7.6)	4 (5.1)
S2	5 (6.3)	6 (7.6)
S2 + 1a	3 (3.8)	2 (2.5)
S3	7 (8.9)	10 (12.7)
S3 + S1a	2 (2.5)	5 (6.3)
S6	10 (12.7)	7 (8.9)
S8	2 (2.5)	4 (5.1)
S9	5 (6.3)	3 (3.8)
S8 + 9	1 (1.3)	4 (5.1)
S10	3 (3.8)	1 (1.3)
Left lung		
S1 + 2	6 (7.6)	6 (7.6)
S1 + 2c	4 (5.1)	5 (6.3)
S3	2 (2.5)	3 (3.8)
S1 + 2 + 3	3 (3.8)	2 (2.5)
S4 + 5	5 (6.3)	7 (8.9)
S6	8 (10.1)	4 (5.1)
S8	4 (5.1)	1 (1.3)
S9 + 10	2 (2.5)	3 (3.8)
S8 + 9 + 10	1 (1.3)	2 (2.5)

### Perioperative results

The surgical data of the two groups of patients after PSM are specified in Table 3. Both groups of surgeries were completed successfully. Three patients in the 3D reconstruction group failed to locate the nodules, mainly because of the deeper nodule location and the difficulty in assessing the nodule location after pulmonary atrophy; four patients in the Hook-wire group failed to locate the nodules mainly because of the Hook-wire falls off. Two cases of pneumothorax, three cases of hemothorax, and four cases of decoupling occurred in the Hook-wire group; no complications of pneumothorax, hemothorax, and decoupling occurred in the 3D reconstruction group. Compared with the Hook-wire group, the 3D reconstruction group has shorter operative time (*P* = 0.001), less intraoperative bleeding (*P* < 0.001), less total postoperative chest drainage (*P* = 0.003), shorter postoperative tube placement time (*P* = 0.001), shorter postoperative hospital stay (*P* = 0.026), and postoperative complications (*P* = 0.035). There was no statistically significant difference between the two groups in terms of pathological type, TNM staging, and number of lymph node dissection.

**Table 3:** Surgical data of patients (cases/$\stackrel{\mathrm{-}}{\text{x}}$ ± *s*)

Characteristic	3D reconstruction group (*n* = 79)	Hook-wire group (*n* = 79)	*P*
Localization failure	3 (3.8)	4 (5.1)	0.699
Pneumothorax	0	2 (2.5)	
Hemothorax	0	3 (3.8)	
Unhook	0	4 (5.1)	
Operative time (min)	117.72 ± 18.02	128.16 ± 21.94	0.001
Intraoperative bleeding (ml)	40.19 ± 10.99	50.57 ± 13.91	< 0.001
Number of lymph node dissection	7.54 ± 2.31	7.27 ± 2.26	0.445
Total postoperative chest drainage (ml)	401.27 ± 40.93	422.03 ± 45.78	0.003
Postoperative tube placement time	3.35 ± 1.73	4.41 ± 1.96	0.001
Postoperative hospital stay (d)	5.25 ± 1.10	6.37 ± 1.15	0.026
Postoperative complications			0.035
Postoperative pulmonary air leak ≥ 3 days	1 (1.3)	3 (3.8)	
Pulmonary infection	4 (5.1)	7 (8.9)	
Thoracic effusion	1 (1.3)	5 (6.3)	
Pathological type			0.769
Benign nodule	2 (2.5)	4 (5.1)	
AAH	5 (6.3)	3 (3.8)	
AIS	20 (25.3)	22 (27.8)	
MIA	34 (43.0)	36 (45.6)	
IAC	18 (22.9)	14 (17.7)	
TNM stage			0.316
Tis stage	14 (17.7)	16 (20.3)	
IA1 stage	43 (54.4)	49 (62.0)	
IA2 stage	22 (27.9)	14 (17.7)	

*Abbreviations*: *AAH* atypical adenomatous hyperplasia, *AIS* adenocarcinoma in situ, *MIA* microinvasive adenocarcinoma, *IAC* invasive adenocarcinoma

## Discussion

In recent years, the resection of lung segments emerged as an effective treatment option for early detection of pulmonary nodules, but the complex anatomy and variability of lung segments make precise resection of lung segments more difficult [4, 5]. It is extremely difficult to find intraoperatively for deeply located, smaller, subsolid nodules [6, 7]. It became a hot topic of research for thoracic surgeons about the appropriate methods of removing lung segments precisely and effectively and improving the successful rate and safety of surgery. In this study, our objective was to investigate the potential clinical benefits of utilizing preoperative 3D reconstruction with CT-guided Hook-wire localization in the treatment of pulmonary nodules.

The CT-guided Hook-wire pulmonary nodule localization method involves inserting a steel needle visible to the naked eye around the pulmonary nodule, reading the chest CT after localization, and determining the location of the Hook wire and the pulmonary nodule to achieve rapid intraoperative location of the lesion. However, it is an invasive operation that can lead to complications such as pneumothorax, hemothorax, and decubitus. Two cases of pneumothorax occurred in the Hook-wire group in this study, which is probably related to the patient’s emphysema and repeated punctures. As a small amount of pneumothorax, neither of which was treated specifically. Previous literature reported that the incidence of pneumothorax was approximately 35% [8], which is asymptomatic and usually does not require treatment. We have thoracentesis kits available in the CT puncture room, and once a large pneumothorax is confirmed, closed chest drainage is performed promptly. Three cases of hemothorax occurred in the Hook-wire group, which presumably results from the injury to the intercostal vessels and pulmonary peripheral vessels during the puncture route. The thin Hook-wire puncture needle, along with thin intercostal and peripheral pulmonary vessels, increases the risk of such bleeding. We performed the puncture within 2 h before the procedure, and the bleeding time was short, and the bleeding caused by the puncture was small, so no special treatment was needed. If shock manifestations such as rapid heart rate and low blood pressure occur, promptly transfuse blood and send to the operating room for resuscitation. Four cases of uncoupling occurred in the Hook-wire group. It is mainly because the pulmonary nodules were close to the pleura and the barb end did not enter the lung completely when the wire was released during CT-guided Hook-wire puncture, resulting in the inability to open the barb. In this situation, the surgeon adds an appropriate amount of sterile water to the chest cavity, instructs the anesthesiologist to drum the lung, identifies the pinhole on the pleural surface, and successfully determines the location of the pulmonary nodule by the position of the Hook-wire pinhole. No complications such as pneumothorax, hemothorax, or decortication occurred in the 3D reconstruction group in this study. This localization method confirms the location of the pulmonary nodule under direct vision during procedure. There is no invasive manipulation during localization and no damage to the intrathoracic structures. To avoid tumor dissemination caused by puncture needles, additional costs associated with invasive procedures and patient pain and anxiety due to puncture positioning. For lung nodules in special locations, such as the pulmonary apex and scapular region, it is more difficult to localize them using Hook wire due to the obstruction of important blood vessels, nerves, and scapulae in the chest wall. However, these specifically located pulmonary nodules have more anatomical landmarks (marker points) on the lung surface. Therefore, the 3D reconstruction pulmonary nodule localization method has unique advantage of specifically located pulmonary nodules.

Three patients in the 3D reconstruction group in this study failed localization, mainly because of severe adhesions in the chest cavity, and the anatomical marker lines (marker points) on the lung surface were destroyed when separating the adhesions. We performed a complete surgical resection of the pulmonary nodule using finger touch as a complementary method. In the case of pleural adhesions, the anatomical position of the lung is altered, and bleeding leads to blurred visualization, making it difficult to distinguish the anatomical landmarks (marker points) on the lung surface. There are relatively few anatomical markers (marker points) on the lung surface in the ribbed surface of the lower lung, making localization difficult using this method [9]. So careful preoperative interpretation of the location of the pulmonary nodule is required. And for pulmonary nodules in this location, CT-guided Hook-wire pulmonary nodule localization is recommended to facilitate rapid intraoperative location of the pulmonary nodule.

This study found that the 3D reconstruction group has significantly shorter operative time, less intraoperative bleeding, less total postoperative chest drainage, and shorter postoperative hospital stays than the Hook-wire group. By studying 3D reconstructed images, surgeons can accurately understand the 3D spatial structure of blood vessels and bronchi before surgery and identify anatomical variants beforehand. Compared to the normal pattern of vascular and bronchial distribution, 3D reconstruction allows earlier evaluation and avoids dissecting more unnecessary lung tissue. Although no anatomical variant vessels were found in the 3D reconstruction group in this study, the target vessels and trachea could be more accurate identification preoperatively, which shortened the intraoperative recognition time of the structures and provided a good assessment of the vascular alignment. It effectively reduces the probability of inadvertent injury and disconnection of intrapulmonary vessels and trachea; reduces excessive freeing of tissues, smaller surgical invasion; and very well shortens the operation time.

Other methods for pulmonary nodule localization include CT-guided coil placement and medical adhesive localization [10]. Spring coil localization is a simple, quick, and highly accurate method but may lead to complications such as lung infection or bleeding during the retention process [11]. Medical adhesive can rapidly solidify inside the body to ensure accurate positioning while also blocking cut blood vessels and promoting blood coagulation, thereby reducing air leakage and bleeding caused by puncture. However, medical adhesive has some drawbacks, including its pungent odor, which can cause discomfort such as coughing in patients. If injected too quickly, it may even cause pulmonary embolism [12]. With the development of digital medical imaging technology, 3D reconstruction techniques have been widely used in various aspects of preoperative assessment of thoracoscopic precision lung segment resection, localization of lung nodules, simulation of surgical protocols, and intraoperative guidance for identification of bronchi and pulmonary vessels [9, 13, 14]. In the era of medicine that emphasizes individualized treatment, preoperative 3D reconstruction images can be used to quickly and intuitively identify individual anatomical patterns through a 360-degree view and observation and judgment from multiple levels and angles, which is an important guarantee for accurate lung segment resection. Compared with CT-guided Hook-wire localization, 3D reconstructed images are cheaper and easier for patients to accept. However, there are limitations to 3D reconstruction guiding surgery. For instance, the lung on the preoperative chest CT scan may be distended and in a normal position, while the lung on the operative side collapses and retracts during thoracoscopic surgery. Therefore, the path of bronchial and vascular travel in the lung segment differs between the two conditions, which requires experience to accurately identify. In addition, 3D reconstruction needs to be completed by surgeons and radiologists who are skilled in the application of relevant software.

This study has certain drawbacks and shortcomings: (I) because the data source included in the study is a single center with limited sample size results and is a retrospective study, which may lead to bias, and (II) the study focused on short-term clinical outcomes in the perioperative period. Hence, further long-term follow-up reviews are necessary to compare and analyze the differences in long-term clinical outcomes between the two groups.

## Conclusion

In summary, CT 3D reconstruction preoperative localization for anatomical lung segment resection in pulmonary nodules not only ensures good localization but also avoids trauma caused by Hook-wire puncture localization. It shortens operation time, reduces intraoperative bleeding, and facilitates rapid postoperative recovery of patients. In addition, it allows the surgeon to clarify the distribution of lung segment structures before surgery, improving the safety of the operation. This approach is worthy of further promotion and application in clinical practice.

## Data Availability

The datasets used and/or analyzed during the current study are available from the corresponding author on reasonable request.

## Abbreviations

CT: Computed tomography
PSM: Propensity score matching
BMI: Body mass index
LUL: Left upper lobe
LLL: Left lower lobe
RUL: Right upper lobe
RML: Right middle lobe
RLL: Right lower lobe
AAH: Atypical adenomatous hyperplasia
AIS: Adenocarcinoma in situ
MIA: Microinvasive adenocarcinoma
IAC: Invasive adenocarcinoma
